# Determination of Biochemical Composition in Peach (*Prunus persica* L. Batsch) Accessions Characterized by Different Flesh Color and Textural Typologies

**DOI:** 10.3390/foods9101452

**Published:** 2020-10-13

**Authors:** Sara Serra, Brendon Anthony, Andrea Masia, Daniela Giovannini, Stefano Musacchi

**Affiliations:** 1 Department of Horticulture, Tree Fruit and Research Extension Center (TFREC), 1100 N. Western Avenue, Washington State University, Wenatchee, WA 98801, USA; sara.serra@wsu.edu (S.S.); brendon.anthony@wsu.edu (B.A.); 2 DipSA—Alma Mater Studiorum, University of Bologna, viale Fanin 46, 40127 Bologna, Italy; andreamasia48@gmail.com; 3 Research Centre for Olive, Tree and Citrus Crops, CREA—Council for Agricultural Research and Economics, Forlì, Via la Canapona 1 bis, 47121 Forlì, Italy; daniela.giovannini@crea.gov.it

**Keywords:** antioxidants, polyphenol oxidase (PPO) activity, nutraceutical properties, polyphenols, red flesh, blood flesh

## Abstract

The rising interest in beneficial health properties of polyphenol compounds in fruit initiated this investigation about biochemical composition in peach mesocarp/exocarp. Biochemical evaluation of phenolic compounds and ascorbic acid were quantified through high-performance liquid chromatography (HPLC) in relation to three flesh colors (white, yellow and red) and four flesh typologies (melting, non-melting, slow softening and stony hard) within six commercial cultivars and eight breeding selections of peach/nectarine in 2007. While in 2008, quality and sensorial analyses were conducted on only three commercial cultivars (‘Big Top’, ‘Springcrest’ and ‘Ghiaccio 1’). The red flesh selection demonstrated the highest levels of phenolic compounds (in mesocarp/exocarp) and ascorbic acid. Total phenolic concentration was approximately three-fold higher in the exocarp than the mesocarp across all accessions. Breeding selections generally reported higher levels of phenolics than commercial cultivars. Flesh textural typologies justified firmness differences at harvest, but minimally addressed variations in quality and phenolic compounds. Flesh pigmentation explained variation in the biochemical composition, with the red flesh accession characterized by an abundancy of phenolic compounds and a high potential for elevated antioxidant activity. Sensorial analyses ranked the cultivar with high soluble solids concentration:titratable acidity (SSC:TA) and reduced firmness the highest overall. Red flesh is a highly desirable trait for breeding programs aiming to improve consumption of peaches selected for nutraceutical properties.

## 1. Introduction

It has become increasingly and consistently clear in the last few years that the composition of secondary metabolites has a notable influence on the quality and nutraceutical properties of foods [1,2]. Among these secondary metabolites, phenolic compounds are the main source of antioxidants, vitamin C and carotenoids in peaches [3]. These compounds are reported to be important in the human diet because they can exert a protective effect against oxidative stress, cardiovascular disease, certain kinds of cancerous tumors and diseases linked to aging [4,5,6,7]. Polyphenols showcase antioxidant properties that can combat oxidative stress, reduce inflammation, and scavenge and reduce free-radicals implicit in many diseases [5,6]. Fruits, specifically in the *Prunus* genus, are full of phytochemicals such as carotenoids, anthocyanins, phenolic acids and flavonoids, compounds that are known for preventing cancers and age-related diseases [8]. However, the levels of these phytochemicals are often dependent on the variety [8]. Due to these health benefits and the consumer’s interest in these “functional foods,” an increased interest in the breeding programs to develop new cultivars with improved nutritional composition (i.e., cultivars with high levels of phenolic compounds and antioxidant compounds) has been noted [9,10].

Peaches are nutritionally important, as they are full of phenolic compounds and vitamins (e.g., ascorbic acid; vitamin C) linked with health benefits and antioxidant activity [10] and are one of the most consumed tree fruits worldwide. However, unfortunately, peach consumption is decreasing worldwide due to poor fruit quality and a lack of meeting consumer expectations [11]. This is largely due to not harvesting at optimal maturity, and/or textural detriments that are a result of post-harvest disorders due to poor handling and storage practices [12]. Solutions to these problems include educating consumers on ripening and “ready to eat” fruit, bolstering the supply chain to support the shipment of physiologically mature fruit, and selecting new cultivars that have superior taste, long-storage capability (achieved by various flesh texture typologies), and that are high in total polyphenol concentration [12,13].

Significant advances in “fruit-keeping” potential has been achieved through selective crosses of standard melting flesh (M) peaches with other textural typologies such as: non-melting (NM), slow softening (SS) and stony hard (SH) [13]. Melting flesh peaches (M) are known to soften dramatically during the last phase of ripening, concomitant to the climacteric respiration and ethylene burst [14], although their firmness can range widely. NM fruits lack the final phase of softening and, while losing some firmness during ripening, they demonstrate a rather firm and “rubbery” texture at maturity, making them more suitable to handling and shipment [15,16]. Although NM peaches are mostly intended for processing, some breeding programs are developing NM peach and nectarines with extended skin blush for the fresh market, taking advantage of the better keeping quality of the NM trait [17]. SS texture is a variant of the M texture, allowing the maintenance of a firm texture for an extended period before the onset of the melting phase, hence a prolonged keeping quality of the fruit on the tree and in the post-harvest as compared to typical M textures [18,19,20]. SH peach phenotypes are characterized with a very firm, crispy flesh and much reduced softening at ripening, along with very little to no ethylene production [21,22,23,24]. The SH flesh typology is determined by a single recessive trait [25], inherited independently from M and NM traits [26]. 

As for maturity status, fruits develop their best organoleptic and commercial qualities during the ripening stage, which consists of a series of highly coordinated and genetically controlled physiological and biochemical changes that make a fruit edible [27]. The major variations in fruit quality during ripening are: exocarp pigment accumulation, increase of total soluble solids concentration, decrease of organic acids, volatilization of aromatic compounds and mesocarp softening [28,29]. All of these biochemical processes are critical for optimal fruit quality and enhanced consumer eating experiences, which is integral for increasing peach consumption worldwide. Therefore, determining harvest time and maturity patterns for new peach cultivars, picking them at optimal maturity and quality, and rapidly delivering them to the consumer is critical [11]. 

Genotype, flesh typology and maturity status all impact fruit quality. However, the phenolic composition of the fruit appears to be largely determined genetically and cultivar dependent [3,30]. Gil et al. [3] stated that there is a wide variation of phenolic content and antioxidant capacity across *Prunus* species/cultivars, with no clear trend observed across yellow and white flesh nectarines and peaches. In contrast, Cantin et al. [10] demonstrated higher total phenolic concentration, antioxidant activity and vitamin C in white-fleshed versus yellow-fleshed peaches/nectarines. However, the nutraceutical composition of these fruits is considered low, when compared to red-flesh peaches (and plums) that have been shown to match blueberries (highest among fruits), in regards to antioxidant activity and total phenolic concentration in the fruit [8,31,32]. However, few studies have investigated the phenolic composition of red flesh (i.e., blood flesh) peaches [32].

Overall, many factors contribute to the composition of beneficial compounds to human health, post-harvest performance and consumer preference. These are factors that should be considered by breeders when selecting parentage for crosses. Horticultural research with the incorporation of modern technologies, such as high-performance liquid chromatography (HPLC), can be used to evaluate fruits beyond just their physiological composition and can assess their metabolic contents [33].

Therefore, the aim of this work was to study the biochemical and physiological aspects of commercial cultivars and breeding program selections that represent various flesh colors and textural typologies. The evaluation was conducted with a special focus on secondary metabolites that affect fruit flavor traits and nutritional properties. In 2007, 14 accessions (commercial varieties and breeding selections) were evaluated for biochemical composition such as ascorbic acid and various phenolic compounds, along with the polyphenol oxidase (PPO) activity. In 2008, a sensorial panel was used to evaluate how physico-chemical properties (e.g., firmness, soluble solid concentration and titratable acidity) related to consumer performance in three commercial cultivars with varying flesh textures.

## 2. Materials and Methods

### 2.1. Peach Cultivars and Breeding Selections

In 2007, 14 peach/nectarine cultivars belonging to the Council for Agricultural Research and Economics (CREA) varietal collection (Forlì, Italy) were evaluated. Six of the accessions were commercial cultivars: ‘Big Top’, ‘Ghiaccio 1’, ‘Springbelle’, ‘Springcrest’, ‘Jonia’ and ‘Oro A’, while eight were selections of the CREA breeding program aimed at increasing the keeping and/or the nutraceutical quality of peaches and nectarines: IFF 311, IFF 813, 26.10.124, 25.1.38, 26.20.117, 21.6.116, 21.6.175 and 26.20.127. 

In 2008, only three cultivars were evaluated: ‘Big Top’, ‘Ghiaccio 1’ and ‘Springcrest’ for quality characteristics. 

These accessions contain an array of flesh colors and textural typologies. In respect to flesh color, fruits were either red (25.1.38), white (‘Ghiaccio 1,’ 21.6.116, 26.20.117, 26.20.124, 26.20.127 and IFF 311) or yellow (IFF 813, 21.6.175, ‘Oro A’, ‘Jonia’, ‘Big Top’, ‘Springbelle’ and ‘Springcrest’) (Table 1). In respect of flesh texture, fruit were also classified into four categories as follows: melting (M) (25.1.38, 21.6.116, 26.20.117, 26.10.124, 26.20.127, 21.6.175, IFF 311, ‘Springcrest’ and ‘Springbelle’), non-melting (NM) (‘Oro A’, ‘Jonia’ and IFF 813), stony hard (SH) (‘Ghiaccio 1’) and slow-softening (SS) (‘Big Top’) (Table 1).

### 2.2. Fruit Quality and Sensorial Analysis 

From each accession (14 in total) harvested in 2007, nine fruits were evaluated for flesh firmness and biochemical analysis. Flesh (i.e., mesocarp) firmness (FF, kg) was measured by a digital penetrometer (53205, TR Turoni S.r.l., Forli, Italy) fitted with an 8 mm plunger and operated manually by handle (range: up to 20 kg force). The same operator carried out all the firmness readings to minimize the impact of the “operator factor” due to the manual mode of utilization. Readings from the two opposite sides of equatorial circumference of the fruit, after removing a thin layer of exocarp were acquired and later averaged to obtain the average firmness for each peach (FF, kg). Five mesocarp replications and three exocarp replications were collected from the nine fruit respectively dicing and peeling each tissue for each accession for the biochemical analysis. Samples were then metabolically quenched by freezing them immediately in liquid nitrogen and stored at −80 °C. Each replication ranged from 10 to 15 g for phenolics composition analyses and from 1.5 to 2.0 g for enzymatic assays [34,35]. 

For the three commercial cultivars harvested and evaluated in 2008, fruit quality and sensorial analyses were conducted to primarily evaluate the impact of textural typology on consumer performance. The external and internal fruit quality/color parameters that were evaluated were: exocarp overcolor, FF (kg), soluble solids content (SSC, %) and titratable acidity (TA, g/L of malic acid).

The exocarp overcolor was measured on the fruit skin’s (i.e., exocarp) sun-exposed surface in the lab immediately post-harvest, by using a colorimeter (CR-300, Konica-Minolta, Ramsey, NJ, USA) to measure three different parameters: L (lightness), a* (greenness–redness) and b* (yellowness–blueness). Exocarp hue (h^o^) and chroma (C*) were then calculated per McGuire [36] and Nunez-Delicado et al. [37] as they express color closer to human perception [38]. FF was measured on 10 fruits per accession using the same procedure described for the 2007 samples. SSC was measured by dispensing a drop of the all fruit juiced onto a refractometer (PAL-1; Atago, Optolab, Modena, Italy). Juice samples from each commercial cultivar were analyzed for titratable acidity executing a titration of 20 mL of flesh peach juice with 0.25 N NaOH using a semiautomatic titrator (Compact-S Titrator, Crison, Modena, Italy) as reported previously [39].

For the sensory evaluation, a panel comprised of six expert peach panelists was organized and each panelist agreed on the definition of the main peach descriptors to be evaluated after a group discussion [40]. The following parameters were appraised: firmness, fibrousness, juiciness, sweetness, acidity, aroma level and overall fruit evaluation. Random coded peach slices belonging to the three different cultivars of varying flesh typology and acclimated to room temperature were presented on a white plate to the panelists who were asked to express their liking for each attribute on every sample. The scores, based on a 9-point hedonic scale, ranged from 1 (low and dislike) to 9 (high and extremely like). Between samples, a quick break was introduced and tap water and unsalted crackers were provided to neutralize taste and odor in panelist’s palates. Food safety and ethical standards were followed handling fruit and selecting compliant assessors to carry out the sensorial evaluation. The average score for each attribute was calculated for the three cultivars in trial.

### 2.3. Polyphenol Oxidase (PPO) Activity 

PPO (E.C. 1.10.3.1) activity values were obtained from the average of three biological samples from each accession harvested in the summer of 2007. For enzymatic analysis, an aliquot of 1.5 g of frozen peach mesocarp was ground to a powder in liquid nitrogen. The frozen powder was added to 5 mL of 200 mM, pH 7.0 phosphate buffer, 5 mM Na_2_EDTA (pH 7.0) and 0.1 g of PVPP (Sigma-Aldrich, St. Louis, MO, USA) previously stored on ice. A homogeneous mixture was achieved and kept in ice for 30 min. All samples were centrifuged at 10,000 relative centrifugal force (rcf) for 30 min at 4 °C and supernatants were collected immediately after for polyphenol oxidase (PPO) activity analyses according to literature [41,42,43,44]. Absorbance was measured at 420 nm against a blank mixture. Enzymatic activities values were expressed as unit of enzyme per gram of peach fresh weight [41].

### 2.4. Ascorbic Acid Extraction and High-Performance Liquid Chromatography (HPLC) Quantification

The protocol outlined in Odriozola-Serrano [45] was used for ascorbic acid extraction, with some modifications. The homogenized samples were collected in 13 mL tubes and centrifuged at 22,000 rcf for 15 min at 4 °C. Supernatants were collected and vacuum filtered through Whatman No.1 paper. Vacuum-filtered samples were passed through a Millipore 0.45 µm membrane, placed into vials, and subsequently injected into the HPLC system. 

The standard calibration curve was prepared with L-ascorbic acid ≥99.9% crystalline (Sigma-Aldrich, St. Louis, MO, USA) at five different concentrations. Dilutions were done with milliQ H_2_O (PureLab ultra system, Elga Lab Water, Lane End, UK). Detection was performed at a wavelength of 254 nm. The mobile phase followed an isocratic method with 0.1% formic acid solution. The total flux per minute was 1 mL, the injection volume was 20 µL and the run time per sample was 12 min. The final concentration of ascorbic acid was expressed as mg of the detected compound per 100 g of peach FW [46].

### 2.5. Extraction of Peach Phenolics and Analysis by HPLC

For the extraction of phenolics an aliquot of each sample, 1 g of freeze-dried peach mesocarp was weighed and put into a 2 mL glass vial. All material was ground up with a mixer mill (MM200, Retsch, Gmbh & Co, Germany) to obtain a fine powder. The extraction was carried out in according to Andreotti et al. [47]. After centrifuging at 17,500 rcf for 30 min at 0 °C, supernatants of all samples were collected and pipetted into new vials and stored at 20 °C until further analyses.

Standards were prepared in 100% methanol (MeOH) (Carlo Erba, Cornaredo, Italy) in a concentration equal to 100 μg/mL. The chosen standards were classified as flavan-3-ols (catechin, epicatechin), cinnamic acid (chlorogenic acid), anthocyanins (procyanidin B2, cyanidin-3-galactoside, cyanidin-3-glucoside), and flavanols (quercetin-3-glucoside, quercetin-3-galactoside, quercetin-3-rutinoside) (Sigma-Aldrich, St. Louis, MO, USA; Extrasynthese Lyon, France). 100 µL of each sample were placed into vials equipped with specific adaptors. The total phenolic concentration (TPC) of each accession was calculated based on the sum of each of the groups, plus the unknown compounds detected by HPLC.

The HPLC apparatus used for analyses included a Waters 1525 binary pump, Waters Inline degasser AF, photodiode detector (PDA, Waters 2996), auto-sampler (Waters 717 plus), and Waters Atlantis™ dC18 (5 µm, 4.6 mm × 250 mm) column (Waters Corporation, En Yvelines Cedex, France).

The mobile phase included two solutions made with HPLC-grade solvents (solution A: 50 mL MeOH, 450 mL milliQ H_2_O, 306 µL ortophosphoric acid; solution B: 450 mL MeOH, 50 mL milliQ H_2_O, 34 µL ortophosphoric acid). The total flux per minute was 1 mL with an injection volume of 20 µL. Running conditions were as follows: 0–5 min 2.5% B, 5–10 min 7.5% B, 10–15 min 12.5% B, 15–20 min 17.5% B, 20–30 min 35% B, 30–40 min 55% B, 40–45 min 75% B and 45–50 min 100% B. For each sample, the total running time was 50 min. The column was reconditioned for 10 min in between samples. The results were analyzed using Empower™ 2 software (Waters Corporation, En Yvelines Cedex, France).

Method reliability was evaluated by a R^2^ value obtained from the standard’s calibration curves (5 different concentrations were used to determine the curve). Detection was performed at a wavelength of 280 nm. Compound identification was based on both retention time and ultraviolet (UV) spectra. Quantification was performed with an internal standard method using the integration of 6-methoxyflavone (Sigma-Aldrich, St. Louis, MO, USA) with the elution time set to 49 min. For the non-identified peaks, their concentrations were estimated using chlorogenic acid standard as a reference, since it was the major representative internal standard compound both in peach mesocarp and exocarp [47]. The final concentration of each detected compound was expressed as mg per gram of dry weight (DW).

### 2.6. Statistical Analysis

The effects of flesh characteristics (textural typology and coloration) on fruit quality and biochemical characteristics were evaluated for significance with an analysis of variance (ANOVA) using Proc GLM in Statistical Analysis System software (SAS Enterprise Guide 7.1) (SAS Institute Inc., Cary, NC, USA). The model considered significance at *p* < 0.05 with the type III sums of squares test. Multiple comparison tests were assessed with Tukey HSD or Student Newman-Keuls (SNK) for post-hoc mean separation assigning different letters where the model was significant at *p* < 0.05. Principal component analysis (PCA) was conducted on mesocarp characteristics in JMP (SAS Institute Inc., Cary, NC, USA). Correlation analysis of biochemical and sensorial analysis, along with data visualization was conducted in Prism v8.2.1 for Windows OS (Graph Pad Inc., San Diego, CA, USA). Sensorial data was not included in any other statistical analyses beyond the correlations in Prism, as it was meant to be qualitative data, in order to provide preliminary information on consumer preference for various flesh types.

## 3. Results

### 3.1. Impact of Flesh Color and Textural Typology on Firmness at Harvest 

In 2007, all accessions were harvested by visual assessment of the ground color (i.e., the loss of chlorophyll), and historical harvest time data. Harvest dates for the associated accessions are displayed in Table 1. These fruits were considered “ready to buy” in respect to their maturity status at harvest [48].

**Figure 1:**
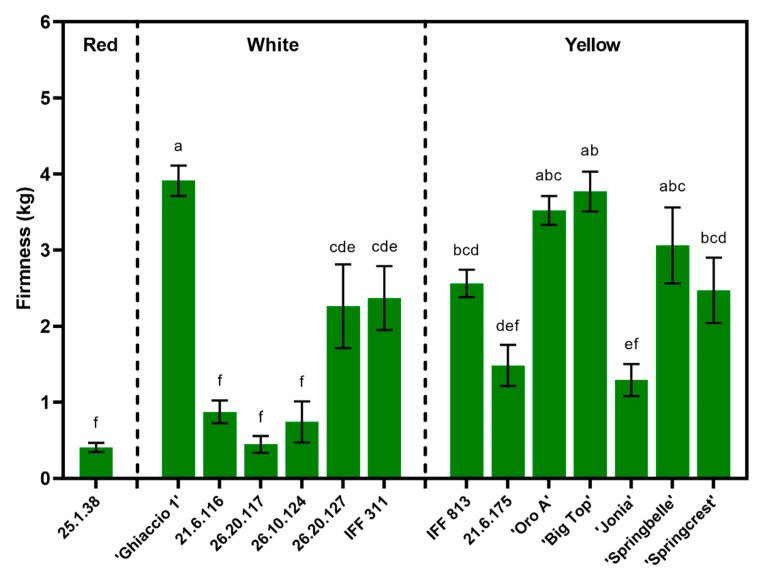
Firmness at harvest in 2007 for all commercial cultivars and breeding accessions divided by flesh color. Student Newman–Keuls (SNK) test used for post-hoc mean comparisons. Columns with the same letter above indicate non-significance at *p* < 0.05. Means ± S.E. displayed.

Average FF values showed great variability across the 14 genotypes, ranging 0.41–3.86 kg (Figure 1). There were minimal differences in FF between white and yellow fleshed accessions (Table 2), while the red flesh, represented by a single accession, 25.1.38, showcased a significantly lower firmness at harvest (Table 2 and Figure 1). Melting flesh (M) and NM accessions demonstrated a similar and reduced firmness at harvest, when compared to the SH and SS accessions (Table 2 and Figure 1). The white fleshed, SH accession ‘Ghiaccio 1’, had the highest firmness at harvest, while the red flesh, M selection, 25.1.38, had the lowest (Figure 1). Several yellow fleshed cultivars demonstrated similar firmness values at the time of harvest (Figure 1).

### 3.2. Impact of Flesh Characteristics on Peach Polyphenol Oxidase (PPO) 

**Figure 2:**
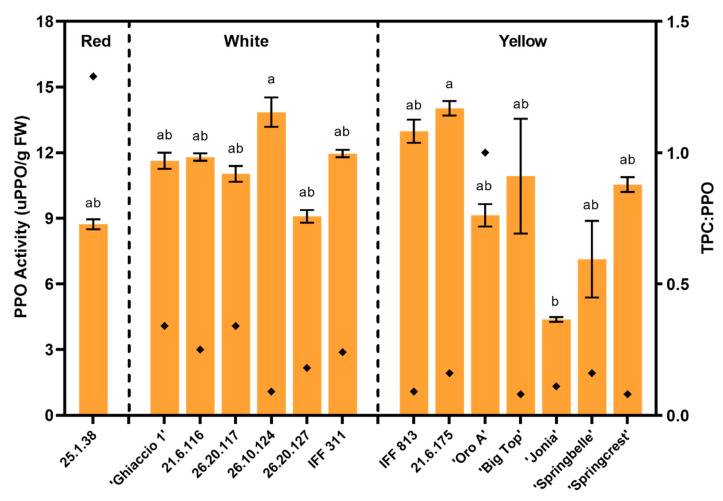
Polyphenol oxidase (PPO) activity (orange columns, primary *y*-axis) and the ratio of total phenolic concentration (TPC):PPO (black diamonds, secondary *y*-axis) at harvest in 2007 for all commercial cultivars and breeding accessions divided by flesh color. Tukey (HSD) test for post-hoc mean comparisons. Columns with the same letter above indicate non-significance at *p* < 0.05. Means ± S.E. displayed. No statistics are presented for the TPC:PPO ratio.

Neither flesh color nor textural typology revealed a significant relationship in respect to PPO activity in the fruit mesocarp (Table 2). The major differences across accessions were between two breeding selections, 26.10.124 and 21.6.175, the highest in enzymatic activity, and the commercial cultivar ‘Jonia’, reporting the lowest PPO activity (Figure 2). The breeding selections are classified as M and showcased lower firmness levels at harvest, while ‘Jonia’ is a NM variety, but did not differ in its firmness when compared to the breeding selections with elevated PPO activity (Figure 1 and Figure 2). There were no other significant differences across accessions (Figure 2). Overall, average values ranged between 4–14 uPPO/g FW across accessions. 25.1.38 and ‘Oro A’ demonstrated high total phenolic concentration (TPC) to PPO activity ratios (Figure 2).

### 3.3. Impact of Flesh Characteristics on Peach Ascorbic Acid Concentration

**Figure 3:**
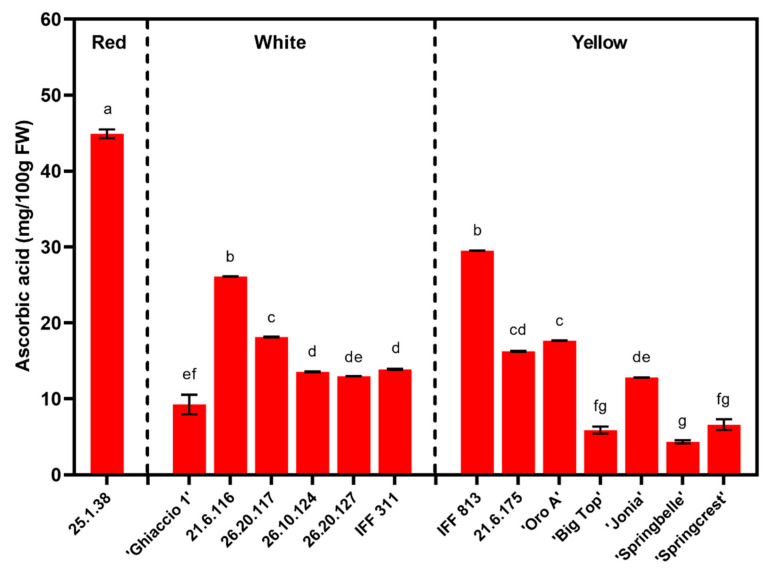
Ascorbic acid at harvest in 2007 for all commercial cultivars and breeding accessions divided by flesh color. Tukey (HSD) test for post-hoc mean comparisons. Columns with the same letter above indicate non-significance at *p* < 0.05. Means ± S.E. displayed.

Ascorbic acid concentration was determined in the mesocarp of the 14 accessions under evaluation in the first year of the trial (Figure 3). Flesh color and textural typology significantly impacted ascorbic acid values (Table 2). The red flesh accession had three to four-fold higher levels of ascorbic acid, when compared to the white and yellow flesh genotypes (Table 2). While, the white flesh accessions had roughly 34% higher values (although not statistically significant) than the yellow (Table 2). A gradient of ascorbic acid values ranged across textural types, in a similar trend as flesh firmness: NM (20.00 mg/100 g FW), M (14.32 mg/100 g FW), SH (9.25 mg/100 g FW) and SS (5.88 mg/100 g FW) (Table 2). 

In general, the breeding selections tended to have much higher ascorbic acid values than the commercial peach cultivars, with extremely elevated levels in the red flesh accession, 25.1.38 (Figure 3). Among the commercial cultivars, ‘Oro A’ had the highest ascorbic acid, while ‘Springbelle’ had the lowest (Figure 3). All of the breeding selections, except for IFF 813 (NM) were classified as M, the same flesh typology as ‘Springbelle’, which had the lowest concentration. ‘Big Top’ and ‘Ghiaccio 1’, classified as SS and SH, were also amongst the lowest ascorbic acid levels. 

### 3.4. Phenolic Concentration and Composition Determined by Flesh Characteristics in Peach Varieties 

Similar to ascorbic acid, total phenolic concentration (sum of all categories evaluated) was significantly higher in the red flesh accession, largely due to anthocyanins and flavanols (Table 2 and Table 3). When compared to white and yellow mesocarp concentrations, red mesocarp concentrations are four to eight-fold higher on average (Table 2). 

In the exocarp, red demonstrates total phenolic concentrations that are five to seven-fold higher than white/yellow cultivars on average (Table 2). The phenolic concentration in the white flesh accessions were also higher than the yellow flesh in the mesocarp, largely due to flavan-3-ol concentrations (Table 2 and Table 3). White and yellow flesh cultivars did not differ in their exocarp total phenolic concentrations (Table 2). The following phenolic compounds were present in the 14 cultivars analyzed: flavan-3-ols, cinnamic acids, anthocyanins, flavonols and unknowns (Table 3). 

In respect to textural properties, SH had the highest mesocarp concentration of phenolics, while the M had the highest in the exocarp, although not significantly different from SH (Table 2). SS had the lowest total phenolic in both the mesocarp and exocarp (Table 2).

**Figure 4:**
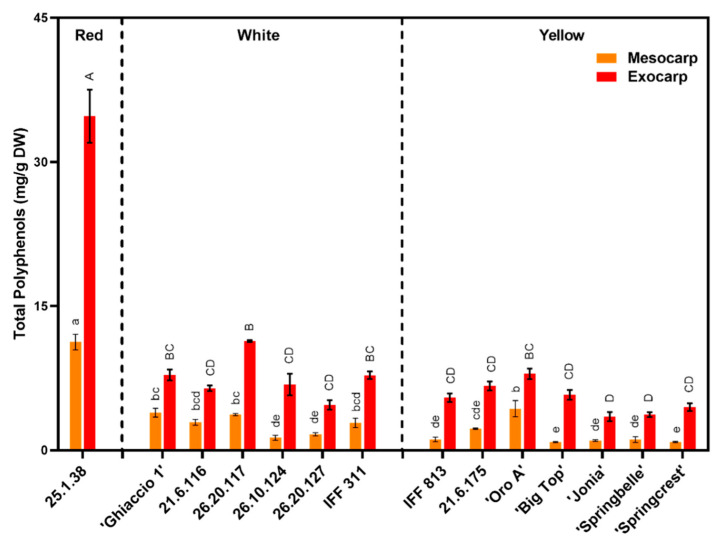
Total phenolic concentration (sum of all categories listed in Table 3) identified in peach/nectarine mesocarp (in orange) and exocarp (in red) tissue sample types across all commercial cultivars and breeding accessions assessed in 2007. Cultivars and accessions are presented divided by flesh color. Tukey (HSD) test for post-hoc mean comparisons. Columns with the same letter above indicate non-significance at *p* < 0.05. Means ± S.E. displayed.

**Figure 5:**
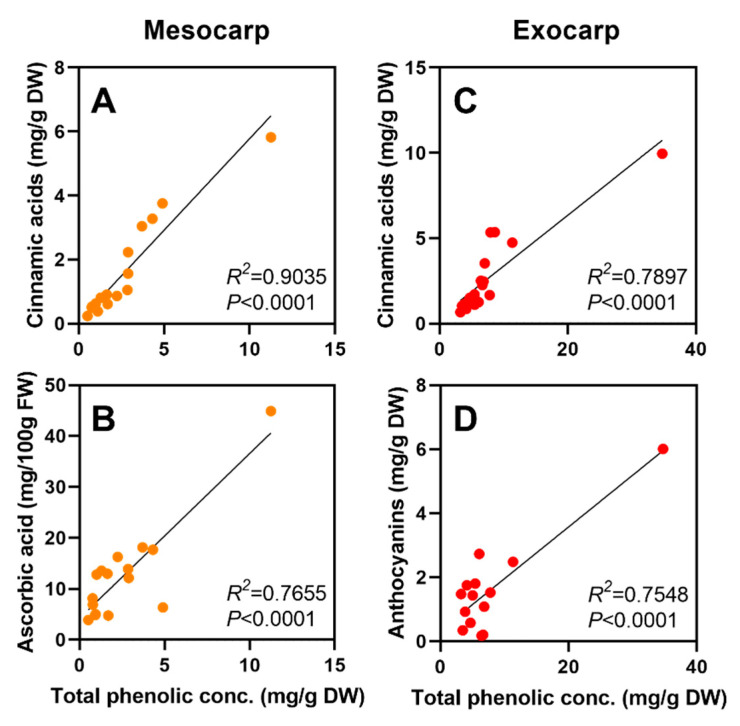
Relationships of total phenolic concentration (TPC) with mesocarp and exocarp characteristics. Cinnamic acids and ascorbic acid demonstrated the highest relationships with TPC across the genotypes in the mesocarp (**A**,**B**). Cinnamic acids and anthocyanins demonstrated the highest relationships with TPC across the genotypes in the exocarp (**C**,**D**).

Although total phenolic concentration showed a high level of variability between mesocarp and exocarp within each accession, the total concentration was always higher (two to four times) in the exocarp across all varieties (Figure 4). The phenolic concentration ratio between exocarp and mesocarp ranged from 1.8 in ‘Oro A’ to 6.8 for ‘Big Top’, with an average of 3.8 across all accessions (data not shown). When considering the different phenolic classes, flavonols and anthocyanins were more present in the exocarp when compared the mesocarp (data not shown). Cinnamic acids and ascorbic acid concentration had strong and significant relationships with the total phenolic concentration in the mesocarp, respectively *R*^2^ = 0.904 and *R*^2^ = 0.766 (Figure 5). While cinnamic acids and anthocyanins had positive relationships with the total phenolic concentration in the exocarp, respectively *R*^2^ = 0.790 and *R*^2^ = 0.755 (Figure 5).

### 3.5. Principal Component Analysis of Biochemical Composition Across Peach Varieties Characterized by Different Flesh Color

**Figure 6:**
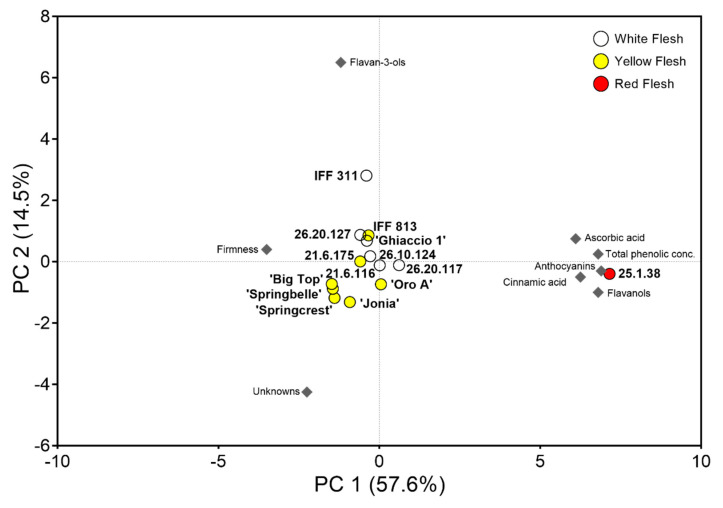
Principal component analysis (PCA) of flesh characteristics on mesocarp quality and phenolic groups. Large circles (scores) visualize the 14 cultivars, colored according to their flesh coloration (yellow, white and red). Grey diamonds indicate the averaged loadings of quality and phenolic data used in the PCA. The scores and loadings are scaled. PCA showcases that flesh coloration (red flesh, in particular) was a major contributor for variation amongst accessions with ~58% of the variation explained on PC1. Additional variation is explained along PC2 (~15%) with flavan-3-ol and unknown compositions appearing to drive vertical separation amongst yellow/white flesh selections.

A principal component analysis (PCA) was conducted to evaluate the separation of accessions based on ascorbic acid concentration, polyphenol composition and flesh firmness. In total, these parameters help explain 72.1% of the variation amongst accessions (Figure 6). PC1 (57.6%) demonstrated the variation as a result of ascorbic acid, firmness and most of the phenolic classes evaluated (e.g., anthocyanins, cinnamic acids, flavanols and the total phenolic concentration) (Figure 6). PC2 (14.5%) explained more of the variation associated with flavan-3-ols and unknown compounds (Figure 6). In general, accessions appeared to cluster based on flesh color, with the red flesh containing a high level of ascorbic acid and TPC, while the white flesh accessions contain higher levels of flavan-3-ols in the mesocarp. Separation was less clear between the white and yellow flesh accessions, although they did appear to cluster based on coloration, except for the yellow fleshed IFF 813 that grouped with the white fleshed. Commercial cultivars and breeding selections were also shown to cluster closer together, except for ‘Ghiaccio 1’ that grouped with the breeding selections. Throughout each parameter evaluated in 2007 (except for PPO), 25.1.38, registered either the highest (in secondary metabolites) or lowest (in firmness) values (Figure 1, Figure 3 and Figure 4), which was confirmed and depicted here in the PCA with such extreme separation from the other accessions (Figure 6).

### 3.6. Sensorial and Fruit Quality Analyses for Commercial Cultivars of Varying Textural Typologies

**Figure 7:**
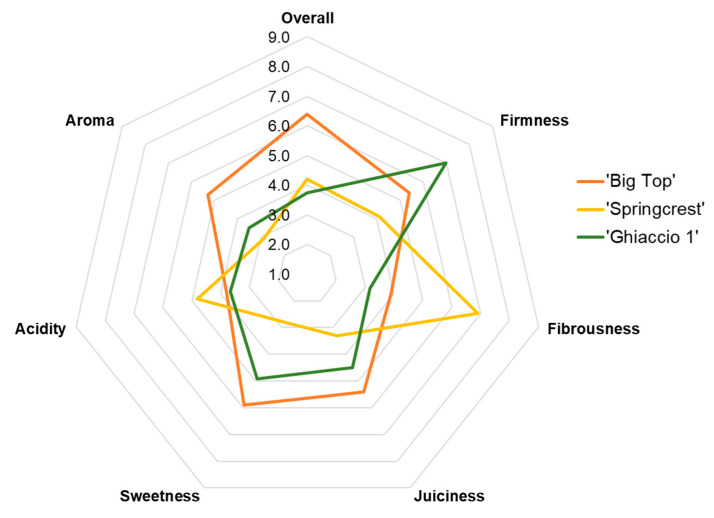
Radar plot of sensory evaluation survey on selected commercial cultivars assessed in 2008. Cultivars include ‘Big Top’ (slow softening, orange), ‘Springcrest’ (melting, yellow) and ‘Ghiaccio 1’ (stony hard, green). A trained panel assessed seven attributes of three commercial cultivars. Attributes assessed include the perception of firmness, fibrousness, juiciness, sweetness, acidity, aroma and an overall evaluation by panelists. The values ranged from 1 (low and dislike) to 9 (high and extremely like). The averages for each cultivar’s evaluation are displayed.

In 2008, sensorial and fruit quality analyses were focused only on ‘Big Top’ (SS—yellow flesh), ‘Ghiaccio 1’ (SH—white flesh) and ‘Springcrest’ (M—yellow flesh) (Figure 7). There was no NM cultivar available in 2008, due to adverse environmental conditions (i.e., hail). 

The sensory panel’s overall fruit evaluation clearly showed that ‘Big Top’ (SS) was the preferred commercial cultivar evaluated by the panelists (Figure 7). ‘Springcrest’ (M) and ‘Ghiaccio 1’ (SH) scored similarly and about two points lower than ‘Big Top’ for their overall evaluations (Figure 7). ‘Ghiaccio 1’ scored highest on the firmness category, while ‘Springcrest’ scored the highest on fibrousness and acidity. In respect to sweetness and juiciness, ‘Big Top’ and ‘Ghiaccio 1’ tend to report higher scores when compared to ‘Springcrest’ (Figure 7). Panelists rated the acidity perceived in ‘Springcrest’ slightly more, in respect to the other two cultivars (Figure 7). 

**Figure 8:**
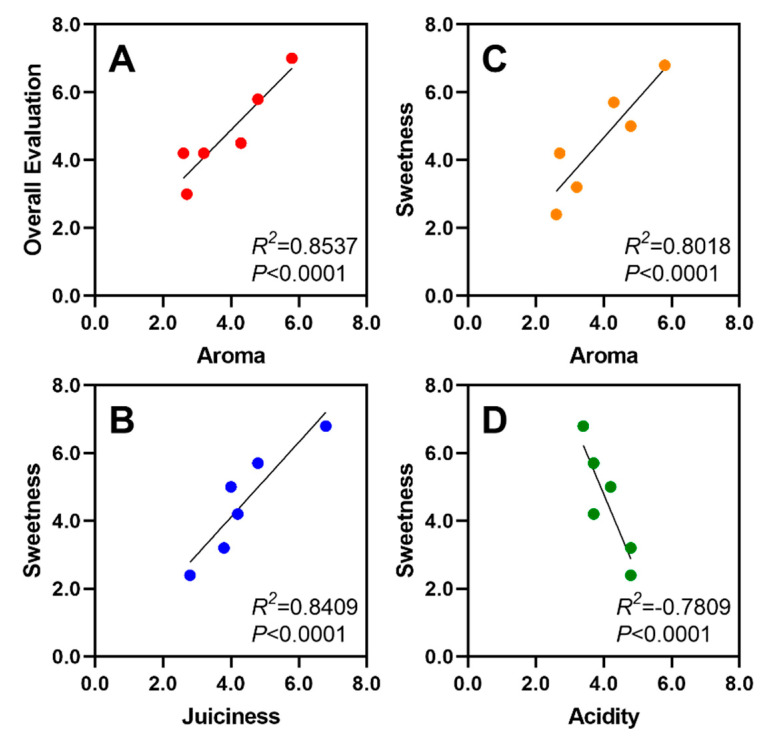
Selected strong relationships between sensory attributes from commercial cultivars evaluated in 2008. Overall evaluation and aroma demonstrate the strongest relationship across sensory data (**A**). Sweetness demonstrated positive relationships with juiciness and aroma (**B**,**C**). Inversely, sweetness exhibits a negative relationship with acidity (**D**).

In respect to the sensorial attributes evaluated, aroma appeared to be the most linked to the overall evaluation score, with a high relationship between these two parameters (Figure 8A). Sweetness appeared to be strongly (*p* < 0.05) and positively correlated to aroma and juiciness (Figure 8B,C), while negative relationship was found with acidity (Figure 8D).

**Figure 9:**
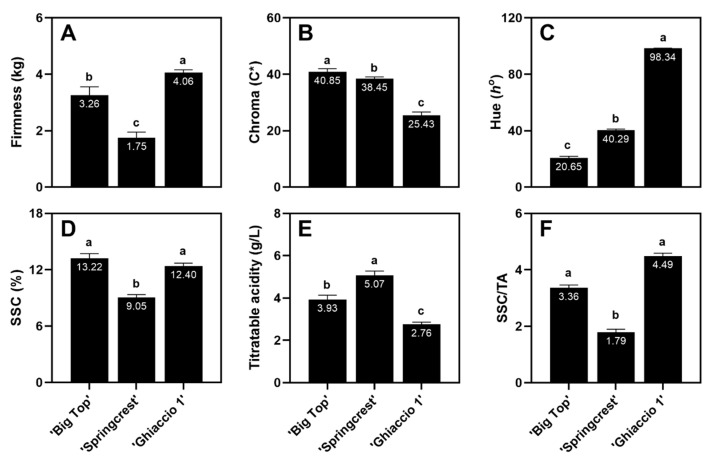
Quality characteristics (firmness (**A**), exocarp chroma (**B**), and exocarp hue angle (**C**), soluble solids concentration (SSC) (**D**), titratable acidity (TA) (**E**), and SSC/TA ratio (**F**)) of the selected commercial cultivars, ‘Big Top’, ‘Springcrest’ and ‘Ghiaccio 1’ at harvest in 2008. Tukey (HSD) test for post-hoc mean comparisons. Columns with the same letter above indicate non-significance at *p*-value < 0.05. Mean comparisons evaluated for each fruit quality characteristic. Means ± S.E. displayed.

Results from fruit quality analyses in 2008 confirm the trend in flesh textural typologies with ‘Ghiaccio 1’, a SH cultivar demonstrating the highest firmness, then ‘Big Top’, a SS nectarine, followed by the lowest, a M flesh variety, ‘Springcrest’ (Figure 9A). Exocarp chroma and hue values are inverted across cultivars, with ‘Ghiaccio 1’ having the highest hue angle evaluated, along with the lowest chroma value, while ‘Big Top’ reported a low hue and a higher chroma in agreement with the clear distinguished overcolors of the two varieties (Figure 9B,C). ‘Big Top’ registered the highest SSC values, although not significantly different from ‘Ghiaccio 1’ (Figure 9D). ‘Springcrest’ reported SSC levels lower than both SS and SH cultivars (Figure 9D). An inverse trend was noted in the TA across cultivars, with ‘Springcrest’ being more acidic at harvest than the other two varieties (Figure 9E). A SSC/TA ratio was calculated, revealing a similar in trend as for the SSC values (Figure 9D,F).

## 4. Discussion

### 4.1. Firmness at Harvest Appeared to Follow Textural Typology Characteristics

Skin ground color (i.e., the loss of chlorophyll) and historical harvest time data were used to evaluate maturity and to determine the sampling dates for the experimental accessions. Due to the range of flesh texture typologies present in the study, flesh firmness was quite variable across all accessions (Figure 1, Table 2). Ideally, having equal firmness (as a maturity indicator) across accessions would allow for better comparisons of quality and nutraceutical properties [11]. However, comparisons between M typologies (softening dramatically until total melting during ripening) [14], SS (softening slower than traditional M, but finally melting as well) [49], NM (softening gradually but remaining ‘gummy’, never melting) [14] and SH (softening minimally and remaining ‘crispy’ even at advanced ripening) [21], made it unlikely to achieve equal firmness across accessions in this study. Furthermore, firmness has been largely discussed for its limited accuracy as a maturity index, especially for various flesh texture types [50]. Brovelli et al. [51] suggests that limited softening in NM textures poses a difficulty in using firmness as a maturity index. The same is true for SH cultivars, which are typically assessed for maturity not by FF, but based on the loss of astringency and ground color in the mesocarp, along with chlorophyll degradation in the exocarp.

Red flesh (also known as blood-flesh) [52] accessions are not very common in the roster of peach varieties and represent a new typology of product [32]. The limited information available on historical harvest dates of 25.1.38 selection and the extensive red blush of the skin in this selection interfering on the correct evaluation of the ground color (i.e., the loss of chlorophyll as a signal of fruit ripening) made it difficult to determine the “optimal” sampling time. 

Flesh typology appears to explain the differences in firmness between accessions, with M and NM being softer than the SS and SH accessions (Table 2). The SH accession ‘Ghiaccio 1’ exhibited the highest firmness at harvest (Figure 1), while new breeding selections that were classified as M, demonstrated lower firmness levels. Among the breeding selections, IFF 813 showed the highest firmness at harvest, this may be explained by its NM textural typology. Overall, the accessions that maintain higher firmness at ripening can potentially have higher post-harvest storability, quality and shelf-life and be less susceptible to bruising and damaging, leading to a new demand for flesh typologies outside of the typical M classification [26]. 

### 4.2. Polyphenol Oxidase, Phenolic Concentration and Browning Potential Across Accessions 

The enzyme polyphenol oxidase (PPO) utilizes phenolics as a substrate to induce browning reactions in peach fruits [53]. For this reason, fruits with high phenolic concentration may contribute to quicker browning reactions [53]. However, there is a large demand for an increase in food consumption that are rich in bioactive phenols (i.e., “functional foods”), due to their antioxidant activity and role in human health benefits [54]. Therefore, it must be the goal of breeders to develop new accessions that are high in TPC, but low in PPO activity, to create healthy fruits without undesirable browning [53].

No significant differences were found when assessing flesh color and textural characteristics, while minimal differences were detected when comparing all the accessions (Table 2 and Figure 2). ‘Jonia’ exhibited the lowest PPO activity, but total phenolic concentration in this accession was also low (Figure 2 and Figure 4). When looking at the ratio of TPC:PPO activity, the red-flesh 25.1.38 selection and the yellow flesh ‘Oro A’ had the highest values (Figure 2), suggesting these are great potential “functional foods” that offer high nutritional benefits, without the high risk of browning. While ‘Big Top’, ‘Springcrest’ and 26.10.124 showed the lowest TPC:PPO ratios (Figure 2), indicating reduced potential antioxidant benefits and a greater potential for browning. 

Catechin, a flavan-3-ol, and chlorogenic acid, a cinnamic acid, are excellent substrates for fruit PPO [7,55]. IFF 311 had elevated levels of flavan-3-ols and high levels of PPO (Table 3 and Figure 2). Similarly, selection 26.20.117 had elevated levels of cinnamic acids and PPO, as well (Table 3 and Figure 2). The elevated levels of these particular phenolic compounds that are associated with PPO substrate indicate a larger potential for browning in these accessions, and may be undesirable for future breeding selections.

### 4.3. Breeding Selections Demonstrated a High Level of Ascorbic Acid Compared to Commercial Cultivars

One of the primary benefits of consuming fruits is the presence of vitamin C (i.e., ascorbic acid) [56]. Saidani et al. [56] reported ascorbic acid levels ranging from 4.15–14.2 mg/100 g FW across nine yellow flesh peach/nectarine cultivars. A similar range was documented in a study evaluating both yellow/white fleshed peaches and nectarines [3]. This ascorbic acid range is in line with the yellow/white flesh commercial cultivars assessed in our study, with the exception of ‘Oro A’, which stood out for its high ascorbic acid values. However, the yellow flesh breeding selections all surpassed this range, along with some white flesh selections as well (26.20.117 and 21.6.116) (Figure 3). Exceptionally high levels of ascorbic acid were observed in the 25.1.38, red flesh selection (44.91 mg/100 g FW) (Table 2 and Figure 3). This was also confirmed in Aubert and Chalot [32], when comparing red and white fleshed cultivars. Although phenolics contribute more to antioxidant capability than ascorbic acid [3,31], ascorbic acid is still considered to be an important part of the fruit’s nutritional benefits. 

Traditionally, peach fruit has been characterized by low ascorbic acid, which averages approximately 7 mg/100 g fresh weight (FW), compared to 60 mg/100 g FW or 50 mg/100 g FW in strawberry and orange, respectively [57]. Although breeding programs are working to enhance this trait, along with other nutraceutical properties in stone fruit [58,59]. A genetic breeding program at New Jersey University (NJU), identified a selection of nectarine, NJN554709, which is characterized by high ascorbic acid content; double that of the average of peach cultivar [57]. While its size, blush and flesh firmness are inadequate for commercial quality, the NJN554709 selection features a particularly good taste and was introduced in the CREA peach breeding program as a parent, to increase the ascorbic acid content and to improve the taste of future accessions [59]. Indeed, the selections 26.20.117, 26.20.124 and 26.20.127 are progenies of crossings between NJN554709 and CREA breeding material of high aesthetics and keeping quality. Other breeding selections present in this study unrelated to NJN554709 showed ascorbic acid values two- to three- times the average value in peach, and in the case of the red-flesh selection 25.1.38, closer to the amount present in oranges (Figure 3). Similarly, Aubert and Chalot [32] reported that red flesh peaches had 40% more ascorbic acid than white accessions. 

In sum, new accessions in this study appear to have superior ascorbic acid levels than pre-existing commercial cultivars, indicating that this breeding program is in fact improving this characteristic in their peach trees, yielding positive results. 

The commercial cultivar, ‘Big Top’, demonstrated one of the lowest levels of mesocarp ascorbic acid in our study (Figure 3), which was also the case in Saidani et al. [56]. In addition to genotypic differences, ascorbic acid levels have also been previously reported to be dependent on maturation stage [60]. Although maturity’s influence on ascorbic acid accumulation appears to be species-specific, with depreciated levels with ripening in apple and mango, and increasing levels with ripening in papaya, apricot and peach [61,62].

In the present study, less firm (and presumably riper) peach accessions like 25.1.38 and 21.6.116 had higher levels of ascorbic acid at harvest (Figure 1 and Figure 3). Aubert et al. [61] affirms this trend of ascorbic acid accumulation in association with peach ripening. This relationship of maturity and vitamin C in peach underscores the significance of harvesting at a more advanced ripening stage in order to achieve the highest nutritional value and quality in fruits, as long as this does not conflict with the need of growers to harvest the fruit safely. In this regard, the long fruit keeping quality of SH genotypes like ‘Ghiaccio 1’ seem to provide the opportunity to combine advanced fruit ripening with good handling resistance [23].

### 4.4. Phenolic Concentration Was Superior in Peach Exocarp and Varied by Flesh Color

The most representative phenolic group in the peach mesocarp in this study were cinnamic acids (Table 3 and Figure 5). In the deanthocyanic cultivar ‘Ghiaccio 1’ (i.e., with no red pigmentation in the skin and the flesh), the anthocyanin class was completely absent (Table 3), similar to Ceccarelli et al. [58]. While in the 25.1.38 selection, characterized by a red flesh, cinnamic acids, anthocyanins and flavonols were much higher than the other accessions (Table 3). Recent literature affirms this trend of red flesh peaches containing higher levels of anthocyanins, cinnamic acids and flavanols when compared to other flesh color varieties [32]. However, flavan-3-ols (in particular catechin), was markedly higher in white flesh accessions [32]. Our study confirms this with flavan-3-ols appearing to be much higher in white flesh accessions (both in the mesocarp and exocarp) (Figure 6 and Table 3). Flavan-3-ols were not detected (zero) in the 25.1.38 mesocarp (Table 3). Overall, selection 25.1.38 had the highest phenolic concentration in mesocarp and exocarp (Figure 4 and Figure 6 and Table 3). 

In general, accessions that had a higher level of red pigmentation (attributable to anthocyanins and flavanols) showed a higher total concentration of phenolic compounds (Table 2 and Table 3 and Figure 4) [32]. Red pigmented stone fruits, like 25.1.38, that contain high levels of phenolic concentration demonstrated high correlations with antioxidant activity and should be incorporated into breeding endeavors to develop “functional” foods to benefit human consumption [9,32]

However, high phenolics are not exclusive to red-flesh fruits. The white fleshed cv. ‘Ghiaccio 1’, along with the breeding selections 26.20.117 (white fleshed) and IFF 813 (yellow fleshed), also showed a relatively high TPC (Figure 4) even without pigmentation compounds in the flesh, due to the high level of cinnamic acids (Table 3). Cinnamic acids also present antioxidant and free-radical scavenging properties [63]. The SH and M textural types demonstrated similar levels of total phenolic concentration in both mesocarp and exocarp (Table 2). This is largely due to ‘Ghiaccio 1’ and 25.1.38 representing two extreme situations in the SH and M typologies. In general, the breeding selections showed higher phenolic concentrations than the commercial cultivars (Figure 4). The NM cultivars tested, all canning peaches with minimal red pigmentation in the skin and in the flesh, along with the SS cultivar ‘Big Top’, had lower phenolic concentrations, with the exception of IFF 813.

IFF 813 also serves as an exception in the PCA clustering (Figure 6), as a yellow fleshed accession clustering in proximity with the white flesh accessions. This accession is classified as deanthocyanic, with no red pigmentation in the mesocarp or exocarp at harvest [17]. Similarly, it had elevated levels of flavan-3-ols, which was attributable to the separation along PC2 (Figure 6). The increased levels of mesocarp flavan-3-ols may also be why the yellow flesh 21.6.175 was closer to some of the white flesh accessions in the PCA (Figure 6). Overall, differences and separation by flesh color was less pronounced between white/yellow accessions [3,30], while dramatic separation was documented between the red flesh accession and white/yellow accessions (Figure 6) [31,32,64]. 

When evaluating phenolic concentration in both peach mesocarp and exocarp, it is evident that the exocarp contains a much higher amount, and the consumption of unpeeled peaches can be seen as a viable nutritional source for human health benefits (Figure 4) [58,65]. Higher phenolic concentrations, along with higher ascorbic acid values have also documented in peach exocarp, when compared to mesocarp [56,66]. This is largely due to the high abundance of anthocyanin concentration in the exocarp (Table 3), which has the potential to contribute to higher levels of antioxidant activity, as previously noted in literature [67]. This positive relationship between TPC and anthocyanins was observed in this present study (Figure 5). Although, flavanols were abundantly present in the exocarp across accessions, the cinnamic acids demonstrated the highest relationship with TPC in both exocarp and mesocarp (Figure 5). 

The red flesh selection, 25.1.38, had the highest levels for all three of these phenolic classes (anthocyanins, flavanols and cinnamic acids) (Table 3). The consistent elevated levels of phenolics (in both mesocarp and exocarp) and ascorbic acid in 25.1.38 highlights once again the significance of this “stand-alone” variety (Figure 6), and its potential superior antioxidant activity and human health benefits. Cultivars like these, especially with high levels of pigmentation, are excellent targets to include in breeding efforts to aid in promoting healthier foods, rich in antioxidants, and increasing peach consumption [10,13].

### 4.5. Sensory Panel Illustrates a Consumer Preference for Cultivars with a High SSC/TA Ratio and Low Firmness

Sensorial and fruit quality evaluations were performed to assess variations amongst different flesh colors and typologies, as well as to survey the general degree of consumer preference across three flesh types. Overall, the preferred cultivar was ‘Big Top,’ perhaps due to its high scores in sweetness and juiciness (Figure 7). ‘Ghiaccio 1’ scored similarly in respect to these two characteristics, and this is perhaps due to their genetic similarity. ‘Big Top’ and ‘Ghiaccio 1’ are both low acid accessions, a trait genetically controlled (D/-), which determines a reduced presence of acids (mainly citric and malic in the ripe fruit) [18,68,69]. The lower acidity (TA) (Figure 9E) could explain much of the higher scores for sweetness perception in ‘Big Top’ and ‘Ghiaccio 1’ when compared to ‘Springcrest’ (Figure 7). In contrast, ‘Springcrest’ is considered a genetically high acid cultivar [65]. This was confirmed with the higher levels of TA (Figure 9E) in ‘Springcrest’, along with the increased perception of acidity by the sensory panel (Figure 7). 

‘Big Top’ reported a perceived sweetness/acidity ratio approximately three times higher than ‘Springcrest’ (Figure 7), along with a quantitative SSC/TA ratio of 3.36 versus 1.79 (Figure 9). This elevated ratio may have played as a strong factor in promoting a higher overall evaluation of ‘Big Top,’ since this ratio has been shown to be a strong predictor for consumer preference in apple [70]. Additionally, the SSC/TA ratio appears to be largely determined by the cultivar [71], hence the variability across accessions evaluated. ‘Ghiaccio 1’ demonstrated a high SSC/TA ratio, but an overall low evaluation by the sensory panel (Figure 7 and Figure 9). This was perhaps due to the higher levels of firmness in this SH variety (Figure 7 and Figure 9).

Furthermore, in respect to perceived firmness, ‘Big Top’ (SS) and ‘Ghiaccio’ (SH) scored similarly (Figure 9). This is supported by a previous result that suggests that at an early stage of the ripening process ‘Big Top’ resembles an SH typology [49,72]. ‘Big Top’ scored high in several categories, including aroma, sweetness, juiciness and overall evaluation (Figure 7). This result may suggest that SS varieties, such as ‘Big Top’, may a superior phenotype with enhanced quality characteristics when compared to other textural types. Although additional varieties and evaluations should be conducted to validate this. In respect to ‘Springcrest’, consumers indicated that the perceived firmness was lower than the other two cultivars, due to its’ M flesh. This melting flesh trait may explain why ‘Springcrest’ scored a higher value in fibrousness, when compared to the harder/crunchier flesh types represented by ‘Big Top’ and ‘Ghiaccio 1’. The exceptional level of fibrousness may have contributed to a reduced overall impression of this variety (Figure 7).

An additional characteristic evaluated during fruit quality analyses of these three commercial cultivars, was the exocarp chroma and hue coloration (Figure 9). Lower hue and higher chroma values were observed in accessions that exhibited a more blushed exocarp (Figure 9). While the high levels of hue and lightness (L* = 78.5; data not shown), along with the low levels of chroma in ‘Ghiaccio 1’ describe well the anthocyianinless nature of this genotype (completely white exocarp and mesocarp). Overcolor coloration did not associate with high-eating quality traits in a previous sensory panel [73]. Regardless, this sensory panel did not evaluate fruit appearance.

Overall, ‘Big Top’, emerged from this analysis as the most sweet, aromatic and juicy cultivar. As a result, ‘Big Top’ was the superior cultivar evaluated, with strong relationships demonstrated across these four parameters in the sensorial analyses (Figure 8). This result suggests that consumers prefer fruits with higher SSC/TA ratios and reduced firmness levels. These quality characteristics can be achieved more easily when fruit are left on the trees longer (i.e., tree-ripe), encouraging advanced maturation and enhanced quality traits for consumer preference [19,74]. However, when conducting fruit quality analyses, sensory panels and biochemical studies to compare various pre-harvest and genetic factors, maturity control should be considered as it influences fruit quality and consumer-perceived characteristics [11].

## 5. Conclusions

In conclusion, this study confirmed the presence of several phenolic compounds in peach, such as flavan-3-ols, cinnamic acids, flavanols and anthocyanins. The total phenolic concentration appears to be largely cultivar dependent, while the subsequent health benefits of these bioactive phenolics are primarily found in the peach exocarp. Mesocarp pigmentation appears to also play a strong role in phenolic determination as well. The red-fleshed breeding selection, 25.1.38, contained the highest amount of ascorbic acid and total phenolic concentration. Flesh color helped explain some of the variation in quality and nutritional benefits found across the 14 peach/nectarine accessions. In the present experimental comparisons, the authors did not identify a specific relationship between the four different flesh typologies and their biochemical profiles. Consumers appeared to prefer ‘Big Top,’ the commercial cultivar that demonstrated higher SSC, lower TA and reduced firmness. Lastly, red flesh accessions may be an ideal consideration to include in stone-fruit breeding efforts in order to bolster the worldwide consumption of antioxidant rich and nutritionally healthy products highly desired by consumers.

## Figures and Tables

**Table 1:** The peach/nectarine commercial cultivars and breeding accessions used for evaluation in the trial with their associated flesh color, textural typology and harvest date in 2007.

Cultivars	Flesh Color	Flesh Typology ^1^	Peach/Nectarine	Harvest Date ^2^
25.1.38	Red	M	Nectarine	09/07/07
‘Ghiaccio 1’	White	SH	Peach	14/07/07
21.6.116	White	M	Peach	25/07/07
26.20.117	White	M	Peach	09/07/07
26.20.124	White	M	Peach	09/07/07
26.20.127	White	M	Peach	25/07/07
IFF 311	White	M	Peach	25/07/07
IFF 813	Yellow	NM	Nectarine	09/07/07
21.6.175	Yellow	M	Peach	25/07/07
‘Oro A’	Yellow	NM	Peach	12/06/07
‘Jonia’	Yellow	NM	Peach	29/06/07
‘Big Top’	Yellow	SS	Nectarine	12/07/07
‘Springbelle’	Yellow	M	Peach	13/06/07
‘Springcrest’	Yellow	M	Peach	19/06/07

^1^ Flesh typology = melting (M), non-melting (NM), slow softening (SS), stony hard (SH) ^2^ harvest date presented as day/month/year.

**Table 2:** The effect of flesh characteristics (color and textural typology) across 14 cultivars on quality and biochemical attributes, such as firmness, PPO, ascorbic acid and total phenolic concentration in the mesocarp and exocarp.

Flesh Characteristics		Firmness (kg)	PPO ^2^ (uPPO/g FW)	Ascorbic Acid (mg/100 g FW)	Total Phenolics (Mesocarp) (mg/g DW)	Total Phenolics (Exocarp) (mg/g DW)
Flesh color	Red	0.41 b	8.73	44.91 a	11.25 a	34.75 a
	White	2.05 a	11.57	14.74 b	2.88 b	7.55 b
	Yellow	2.64 a	9.76	10.99 b	1.43 c	5.15 b
Significance		***	ns	***	***	***
Flesh typology ^1^	M	1.60 b	10.53	14.32 a,b	2.71 a,b	8.63 a
	NM	2.42 b	9.39	20.00 a	2.15 b,c	5.64 b
	SH	3.86 a	11.63	9.25 b,c	3.90 a	7.83 a,b
	SS	3.60 a	9.96	5.88 c	0.85 c	5.76 b
Significance		***	ns	***	**	ns

ns, **, *** indicate no significance or significance at *p* < 0.01 or 0.001. Tukey (HSD) test for mean comparisons; means in columns with the same letter indicate non-significance at *p*-value < 0.05; ^1^ flesh typology = melting (M), non-melting (NM), slow softening (SS), stony hard (SH); ^2^ polyphenol oxidase (PPO).

**Table 3:** Impact of flesh color, typology and sample tissue type (mesocarp and exocarp) on phenolic composition in peach/nectarine commercial cultivars and breeding accessions in 2007. Phenolic group classifications detected include: flavan-3-ols, cinnamic acids, anthocyanins, flavanols and unknown compounds identified with high-performance liquid chromatography (HPLC).

Tissue Type	Genotype	Flesh Color	Flesh Type ^1^	Flavan-3-ols (mg/g DW)	Cinnamic Acids (mg/g DW)	Anthocyanins (mg/g DW)	Flavanols (mg/g DW)	Unknowns (mg/g DW)
Mesocarp	25.1.38	Red	M	-	5.82 a	1.87 a	3.46 a	0.10 e
	‘Ghiaccio 1’	White	SH	0.66 b	2.99 b	-	-	0.22 b,c,d,e
	21.6.116		M	0.77 b	1.56 c	0.04 b,c	0.06 b	0.47 a
	26.20.117		M	0.34 b,c	3.04 b	0.02 c	-	0.25 b,c,d,e
	26.10.124		M	0.20 b,c	0.81 c	0.09 b,c	0.06 b	0.12 d,e
	26.20.127		M	0.53 b,c	0.90 c	0.10 b,c	-	0.12 d,e
	IFF 311		M	1.48 a	1.05 c	0.15 b	0.07 b	0.10 e
	IFF 813	Yellow	NM	0.50 b,c	0.38 c	-	0.10 b	0.15 c,d,e
	21.6.175		M	0.72 b	0.86 c	-	0.26 b	0.40 a,b,c
	‘Oro A’		NM	0.36 b,c	3.28 b	-	0.24 b	0.42 a,b
	‘Big Top’		SS	-	0.54 c	0.05 b,c	-	0.25 b,c,d,e
	‘Jonia’		NM	-	0.63 c	-	-	0.38 a,b,c
	‘Springbelle’		M	0.13 c	0.42 c	-	0.23 b	0.33 a,b,c,d
	‘Springcrest’		M	0.01 c	0.50 c	-	-	0.35 a,b,c
Significance				***	***	***	***	***
Exocarp	25.1.38	Red	M	0.26 g	9.96 a	6.01 a	17.76 a	0.66 b
	‘Ghiaccio 1’	White	SH	2.36 a	4.45 b	-	0.54 b	0.47 b
	21.6.116		M	1.70 b,c,d,e	2.51 c	0.18 e,f	1.17 b	0.88 a,b
	26.20.117		M	1.46 c,d,e	4.74 b	2.48 b	1.93 b	0.75 a,b
	26.10.124		M	1.30 d,e	2.47 c	1.08 c,d,e,f	1.41 b	0.55 b
	26.20.127		M	1.79 b,c,d,e	1.50 c,d	0.58 c,d,e,f	0.44 b	0.41 b
	IFF 311		M	2.23 a,b	1.67 c,d	1.52 b,c,d	1.79 b	0.59 b
	IFF 813	Yellow	NM	2.00 a,b,c,d	1.11 c,d	-	2.01 b	0.81 a,b
	21.6.175		M	2.02 a,b,c	2.26 c	0.20 d,e,f	0.99 b	1.22 a
	‘Oro A’		NM	1.13 e,f	5.35 b	-	1.02 b	0.43 b
	‘Big Top’		SS	0.14 g	1.47 c,d	2.27 b	1.33 b	0.53 b
	‘Jonia’		NM	0.46 f,g	1.04 c,d	0.34 d,e,f	0.87 b	0.77 a,b
	‘Springbelle’		M	0.18 g	0.76 d	1.62 b,c	0.66 b	0.48 b
	‘Springcrest’		M	0.27 g	1.23 c,d	1.18 c,d,e	1.19 b	0.60 b
Significance				***	***	***	***	**

Means displayed; *n* = 3; **, *** indicate significance at *p*-values of <0.01 or 0.001. Tukey HSD test for mean comparisons; means in columns with the same letter indicate non-significance at *p* < 0.05; Tissue types (mesocarp vs. exocarp) were statistically assessed independently of each other; ^1^ flesh typology = melting (M), non-melting (NM), slow softening (SS), stony hard (SH); - indicates a zero-value (undetectable) and were excluded from statistical analyses.
